# Inhaled sphingosine reduces bronchial *Pseudomonas aeruginosa* burden during porcine ex vivo lung perfusion and shows tissue delivery in explanted human lungs

**DOI:** 10.1038/s41598-026-65074-7

**Published:** 2026-08-06

**Authors:** Yongjie Liu, Fabian Schumacher, Yuqing Wu, Lydia Leukers, Kristin Schimank, Omar Abou-Issa, Christian Taube, Andreas Wissmann, Nikolaus Pizanis, Burkhard Kleuser, Achim Koch, Erich Gulbins, Markus Kamler

**Affiliations:** 1 Department of Thoracic and Cardiovascular Surgery, Thoracic Transplantation, West German Heart and Vascular Center, University Hospital Essen, University Duisburg-Essen, Essen, Germany; 2 Institute of Molecular Biology, University Hospital Essen, University Duisburg-Essen, Essen, Germany; 3 Department of Biology, Chemistry and Pharmacy, Institute of Pharmacy, Freie Universität Berlin, Berlin, Germany; 4 Department of Pulmonary Medicine, University Hospital Essen, University Duisburg-Essen, Essen, Germany; 5 Central Animal Laboratory, University Hospital Essen, University Duisburg-Essen, Essen, Germany; 6 Present Address: Department of Transplantation, Department of Thoracic Surgery and Oncology, State Key Laboratory of Respiratory Disease & National Clinical Research Center for Respiratory Disease, The First Affiliated Hospital of Guangzhou Medical University, Guangzhou, 510120 China

**Keywords:** Sphingosine, Bacterial pathogens, Pneumonia, Isolated perfused and ventilated lungs, Lung transplantation, Diseases, Drug discovery, Medical research, Microbiology, Pathogenesis

## Abstract

*Pseudomonas aeruginosa* infection remains a major challenge in donor lung management and post-transplant respiratory care, particularly in the setting of antimicrobial resistance. Ex vivo lung perfusion (EVLP) provides an opportunity for localized antimicrobial intervention before transplantation and allows detailed assessment of infection-associated lung injury under controlled conditions. In this study, we evaluated inhaled sphingosine in a porcine EVLP model of acute *P. aeruginosa* airway contamination using three experimental groups: uninfected controls, infected lungs treated with 0.9% NaCl, and infected lungs treated with sphingosine. This design allowed us to assess both the impact of *P. aeruginosa* infection on EVLP physiology and the effects of sphingosine on bacterial burden and infection-associated lung changes. Infected porcine lungs showed greater declines in dynamic and static compliance than uninfected controls, whereas pulmonary artery pressure, peak airway pressure, oxygen-exchange capacity, lactate accumulation, lung weight gain, and histological injury scores were not significantly worsened under the present experimental conditions. Inhaled sphingosine reduced bronchial *P. aeruginosa* CFU counts, whereas 0.9% NaCl did not. Sphingosine treatment did not further impair lung physiology, oxygenation, lactate accumulation, histological injury, or lung weight gain. Mechanistic analyses showed association of sphingosine with bacterial cardiolipin and increased colocalization of sphingosine with *P. aeruginosa*, consistent with a membrane-associated antibacterial mechanism described in previous studies. In exploratory experiments using four explanted human recipient lungs, sphingosine inhalation increased sphingosine levels in bronchial and parenchymal tissue and altered related sphingolipid metabolites, including sphingosine-1-phosphate, ceramide, and sphingomyelin, without apparent acute histological damage in assessed airway samples. One of the four human specimens showed bacterial colonization, in which a marked reduction in detectable bacterial growth after sphingosine inhalation was observed as a hypothesis-generating finding. Together, these data suggest that inhaled sphingosine can reduce acutely accessible bronchial *P. aeruginosa* burden during porcine EVLP without detectable short-term adverse effects and provide a basis for further investigation of sphingosine-related lipid metabolism and local antimicrobial strategies during EVLP.

## Introduction

*Pseudomonas aeruginosa* (*P. aeruginosa*) is a Gram-negative pathogen commonly associated with various hospital-acquired infections, including healthcare-associated pneumonia, ventilator-associated pneumonia, catheter-associated urinary tract infections, surgical site infections, and bloodstream infections^1^. These infections occur frequently in patients with compromised immune systems, such as patients with tumors, immunosuppression, burns, and cystic fibrosis^1^.

*P. aeruginosa* infection is also a common complication in lung transplantation, where it is a leading cause of ventilator-associated respiratory infections after transplantation and is associated with higher hospital mortality^2,3^. Early control of *P. aeruginosa* infection is important for improving outcomes, especially after lung transplantation^4^. However, *P. aeruginosa* is a known multidrug-resistant pathogen, which can make eradication difficult. It can utilize innate and acquired resistance mechanisms to counteract the effects of many antibiotics, resulting in recurrent and chronic infections^5^. The emergence of multidrug-resistant *P. aeruginosa* strains has become a major cause of morbidity and mortality, particularly in intensive care units and long-term care facilities^6^. Additional therapeutic strategies are therefore needed to address this pathogen after lung transplantation^7^. Numerous innovative therapeutic strategies have been developed to address this issue, including inhibition of quorum sensing and bacterial lectins, iron chelation, phage therapy, vaccination, nanoparticles, antimicrobial peptides, and electrochemical scaffolds^8^.

Another emerging approach is the use of sphingosine, a long-chain amino alcohol that is a component of cell membranes in most eukaryotic organisms and has shown broad antibacterial activity against various bacteria, including *P. aeruginosa*^9–15^. In a recent study, bronchiectasis patients showed markedly decreased sphingosine levels in their airways compared with controls^16^. This reduction was even more pronounced among bronchiectasis patients with *P. aeruginosa* infection, suggesting that diminished airway sphingosine levels may compromise epithelial antibacterial defense and increase susceptibility to *P. aeruginosa* infection^16^. We have recently shown that sphingosine interacts with cardiolipin in the bacterial cell membrane and induces cardiolipin degradation, resulting in bacterial membrane permeabilization and death^17^.

Ex vivo lung perfusion (EVLP) is a technique that allows assessment and reconditioning of marginal lungs previously considered unsuitable for transplantation^18^. EVLP has also been used as a platform to treat infected donor lungs with antimicrobial agents, resulting in reduced bacterial counts and endotoxin levels in bronchoalveolar lavage fluid and improved donor-lung function^19^. Our research group has previously shown that sphingosine can be administered safely in pigs and pig lungs undergoing EVLP^20,21^. We also found that infections of isolated ventilated pig lungs with the laboratory *P. aeruginosa* ATCC 27,853 strain can be treated with inhaled sphingosine^22^.

Importantly, bacterial colonization or contamination of donor lungs may complicate donor acceptance and perioperative management. Therefore, an EVLP-based local antimicrobial strategy could provide an opportunity to reduce airway bacterial burden before transplantation and may support safer utilization of selected marginal donor lungs.

However, previous sphingosine lung-perfusion studies did not include an uninfected EVLP control group. Therefore, they could not systematically determine the extent to which *P. aeruginosa* infection itself affects lung physiology, oxygen-exchange capacity, tissue injury, or edema formation during EVLP. In addition, it remained unclear whether sphingosine treatment only reduces bacterial burden or whether it also modifies infection-associated functional or histological changes during EVLP. These questions are important because an antibacterial strategy for donor-lung reconditioning should ideally reduce bacterial burden without worsening lung function or causing additional tissue injury.

In this study, we investigated inhaled sphingosine in a porcine EVLP model using the clinical *P. aeruginosa* isolate 762^23^. In contrast to previous work, we included three experimental groups: uninfected controls, infected lungs treated with 0.9% NaCl, and infected lungs treated with sphingosine. This design allowed us to evaluate not only the antibacterial effect of sphingosine, but also the impact of acute *P. aeruginosa* airway contamination on EVLP physiology and whether sphingosine treatment modifies infection-associated changes. We therefore performed a detailed assessment of pulmonary artery pressure, dynamic and static compliance, peak airway pressure, oxygen-exchange capacity, lactate accumulation, lung weight gain, and histological injury during EVLP.

Furthermore, to explore the relevance of inhaled sphingosine in human lung tissue, we performed exploratory experiments using explanted human recipient lungs. These experiments were primarily designed to assess tissue delivery of inhaled sphingosine and short-term local tolerability, rather than to establish antibacterial efficacy in human lungs. In addition, we quantified sphingosine-related lipid metabolites, including sphingosine-1-phosphate, ceramide, and sphingomyelin, in bronchoalveolar lavage fluid, bronchial epithelial tissue, and lung parenchyma. These analyses provide preliminary information on how inhaled sphingosine may influence local sphingolipid metabolism in human lung tissue and may serve as a basis for future studies investigating sphingosine-related lipid metabolic pathways during EVLP.

## Materials and methods

All methods were performed in accordance with the relevant guidelines and regulations.

### Human lung experiments

The use of explanted human recipient lung material was approved by Ethikkommission des Universitätsklinikum Essen (West German Heart and Vascular Center Essen, University of Duisburg-Essen, Hufelandstrasse 55, 45,122 Essen, Germany), (No. 17–7326-BO). Informed consent was obtained from all subjects and/or their legal guardian(s). In total, four explanted human recipient lungs obtained at the time of lung transplantation were included in these ex vivo experiments. The underlying indications for transplantation included end-stage lung diseases such as emphysema and pulmonary fibrosis. These human lung experiments were not performed on the XVIVO EVLP system because the explanted recipient lungs had severely diseased vascular structures that were not suitable for connection to a perfusion circuit. Instead, they were performed under ex vivo selective ventilation. For lipid and histological analyses, the right lower lobe was selectively ventilated for 15 min with simultaneous sphingosine inhalation, while the contralateral left lower lobe served as the untreated control and was ventilated for the same duration without sphingosine. BALF collection and tissue sampling were performed using the same protocol in treated and untreated regions. For bronchial epithelial and lung parenchymal lipid analyses, two tissue subsamples were collected from each treated and untreated region of each lung. The measurements from the two subsamples were averaged to obtain one value per lung and condition, with the individual lung serving as the biologically independent experimental unit. BALF and tissue samples were then collected for microbiological assessment, histological analysis, and quantification of sphingosine-related lipids by LC–MS/MS. Bacterial colonization was not known before the experiments. Microbiological assessment identified bacterial colonization in one of the four explanted lungs. In this colonized specimen, BALF from the same treated lobe was collected before and after sphingosine inhalation to assess the antibacterial effect of treatment.

### Animal studies

All animal procedures were conducted under the supervision of and approved by the Central Animal Laboratory of the University of Duisburg-Essen, following the Principles of Laboratory and Animal Care, the Guide for the Care and Use of Laboratory Animals, and the ARRIVE guidelines (Animal Research: Reporting of In Vivo Experiments). Euthanasia was performed in accordance with the Animal Welfare Act (TierSchG).

### Ex vivo lung perfusion

The pigs were initially tranquilized with azaperone (Stresnil) (2 mg/kg) and ketamine (30 mg/kg). Following a 10-min interval, deep anesthesia was achieved through intravenous injection of midazolam (0.5 mg/kg) and ketamine (30 mg/kg). Subsequently, euthanasia was induced by administration of KCl (1.67 ml/kg). After confirming death, heparin (300 IU/kg) was administered to reduce blood clot formation during lung procurement. A median sternotomy was performed and the pig lungs and trachea were isolated. The lungs were flushed anterogradely and retrogradely with 2 L of Perfadex (XVIVO Perfusion, Gothenburg, Sweden) via the pulmonary artery and right atrium, respectively. Afterward, the lungs were placed in a preservation bag filled with Perfadex and stored at 4 °C for 2 h.

The EVLP system (XPSTM, XVIVO Perfusion, Gothenburg, Sweden) was set up in accordance with the manufacturer’s guidelines. The pulmonary artery and left atrium were connected to the perfusion circuit and ventilator. The lung was perfused for 4 h with Steen solution (XVIVO Perfusion, Gothenburg, Sweden) following the XVIVO perfusion guidelines.

No systemic antibiotics were added to the EVLP perfusate during the experimental period, in order to avoid confounding the assessment of the local antibacterial effect of inhaled sphingosine.

### Experimental groups

**Fig. 1:**
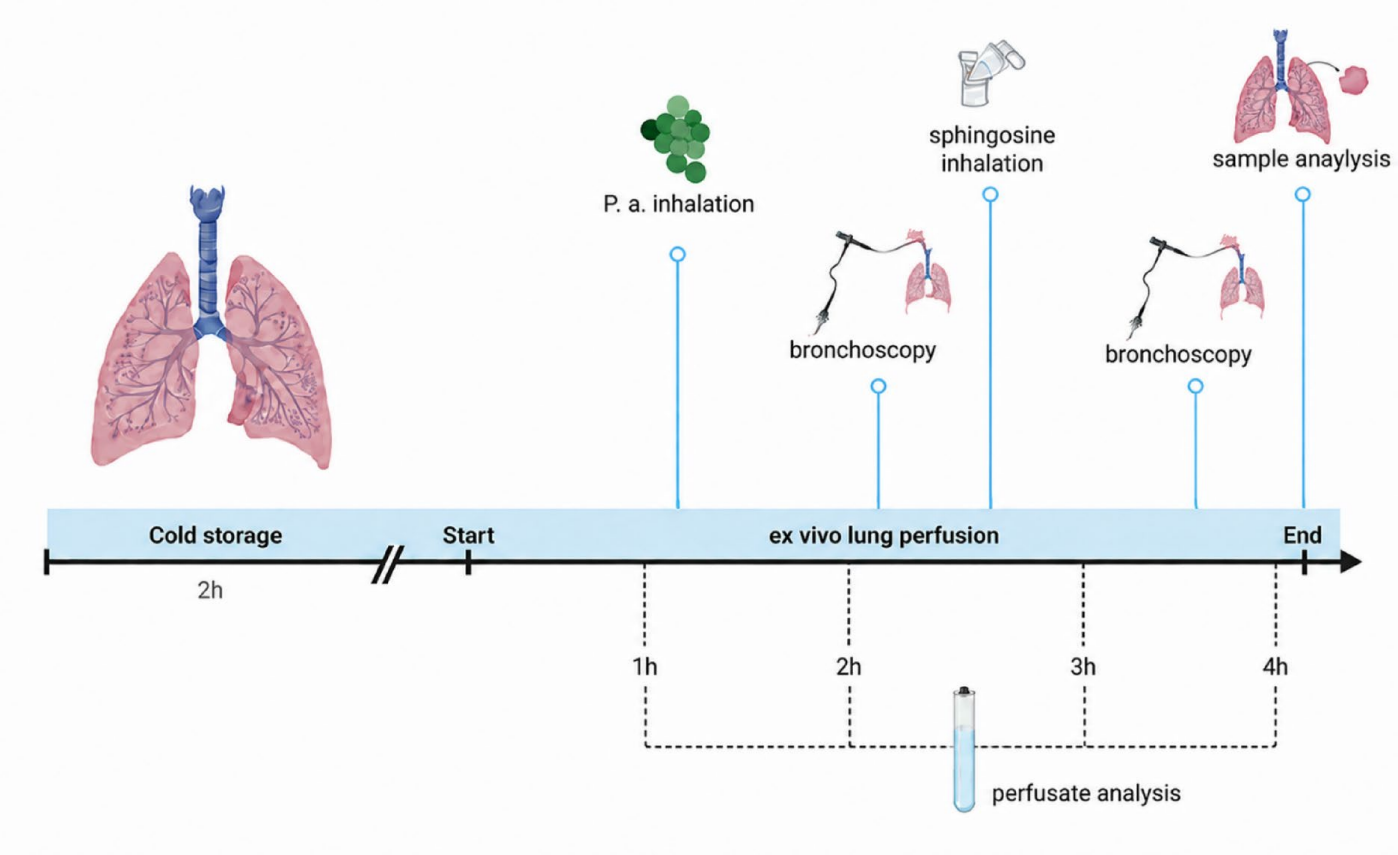
Experimental timeline of the porcine EVLP infection and sphingosine treatment protocol. Porcine lungs were stored at 4 °C for 2 h before initiation of ex vivo lung perfusion (EVLP). After the start of EVLP, lungs were exposed to *P. aeruginosa* by inhalation, whereas uninfected control lungs received 0.9% NaCl inhalation at the corresponding time point. Bronchoscopic biopsies were collected before treatment to determine the bacterial burden. One hour after infection, infected lungs were treated with inhaled sphingosine or 0.9% NaCl, followed by repeat bronchoscopy for post-treatment bacterial quantification. Perfusate samples were collected during EVLP for blood gas and biochemical analyses. At the end of the 4-h EVLP procedure, lung tissue samples were collected for microbiological, histological, and additional sample analyses.

The experimental workflow is shown in Fig. 1. Fifteen experiments were randomly distributed among three cohorts: the uninfected group (n = 5), the *P. aeruginosa* strain 762 infected and 0.9% NaCl treated group (n = 5), and the *P. aeruginosa* strain 762 infected and sphingosine treated group (n = 5). For infected cohorts, the lungs were exposed to *P. aeruginosa* strain 762 via inhalation. Concurrently, the uninfected group received inhalation of 0.9% NaCl solution. One hour after infection, the infected + sphingosine group received sphingosine by nebulization, whereas the uninfected group and the infected + 0.9% NaCl group received 0.9% NaCl at the corresponding time point.

The 1-h interval between bacterial inoculation and treatment was chosen to allow initial airway exposure and interaction of P. aeruginosa with the bronchial mucosa while maintaining a clinically feasible EVLP intervention window.

### Infection and sphingosine treatment

*P. aeruginosa* strain 762 (obtained from a urosepsis patient)^23^ was cultured for 16 h on tryptic soy agar (TSA) plates (Becton Dickinson Biosciences, Germany). The bacteria were subsequently transferred into 40 ml of pre-warmed (37 °C), sterile tryptic soy broth (TSB) (Becton Dickinson Biosciences), and the optical density (OD) was standardized to 0.2–0.25, followed by an incubation period of 60 min at a temperature of 37 °C with constant agitation at a speed of 125 rpm. After one hour, the bacteria were transferred to a 50 ml Falcon tube and centrifuged (10 min, 3000 rpm). The supernatant was removed, and the bacteria were resuspended in 0.9% NaCl to a concentration of 10^9^ colony-forming units (CFU) per 3 mL. Bacteria were inhaled into the isolated perfused lung using a nebulizer (Aerogen Ultra, Aerogen Limited, Galway, Ireland). One hour after infection, sphingosine was sonicated, diluted to 125 μM in 10 mL NaCl, and administered through an ultrasound nebulizer (Aerogen Ultra, Aerogen Limited, Galway, Ireland).

### Quantification of bacteria in bronchi

Bronchial biopsy samples were acquired using a fiber-optic video scope (Ambu A/S, Baltorpbakken 13, DK-2750 Ballerup, Denmark) to quantitatively assess bacterial load in the bronchial epithelium. Sampling was standardized by using the same bronchoscopic forceps and by obtaining pre- and post-treatment biopsies from comparable segmental or subsegmental bronchial sites of the treated lung region whenever technically feasible. These samples were procured before and 60 min after sphingosine or 0.9% NaCl administration and were subsequently subjected to a 15-min incubation period in 1 mL of 5% saponin solution. Subsequently, 50 μL of this solution was carefully streaked onto an agar plate using an inoculation loop. Following overnight incubation, the plate CFUs were enumerated. CFU counts were expressed per biopsy sample rather than normalized to total tissue weight, because biopsy weight may be influenced by variable amounts of cartilage and submucosal tissue in addition to bronchial epithelium. Since the bacterial burden in this airway contamination model was expected to be primarily associated with the bronchial epithelial surface, normalization to total tissue weight may not accurately reflect the epithelial area sampled.

### Lung function monitoring during EVLP

During the 4-h EVLP procedure, physiological lung parameters, including pulmonary artery pressure (PAP), dynamic compliance (Cdyn), static compliance (Cstat), and peak airway pressure (Ppeak), were recorded using the perfusion system. To evaluate the effects of *P. aeruginosa* infection and subsequent sphingosine inhalation, repeated measurements were averaged within predefined time intervals for each individual lung. Changes between the corresponding pre- and post-intervention intervals were then calculated for each lung. ΔPAP1, ΔCdyn1, ΔCstat1, and ΔPpeak1 represent changes in mean values after the first intervention, namely *P. aeruginosa* inhalation in the infected groups or 0.9% NaCl inhalation in the uninfected control group. ΔPAP2, ΔCdyn2, ΔCstat2, and ΔPpeak2 represent changes in mean values after the second intervention, namely sphingosine inhalation in the infected + sphingosine group or 0.9% NaCl inhalation in the uninfected and infected + 0.9% NaCl groups. These per-lung change values, rather than individual time-point measurements, were used as the statistical unit. Comparisons of changes between independent experimental groups were performed using unpaired two-tailed t-tests.

To evaluate oxygen-exchange function, the lung was ventilated with 100% oxygen every hour for 10 min. Perfusate samples from both the pulmonary artery and vein were subjected to hourly analysis using a blood gas machine (LUMIRATEK®i15, LumiraDx, Kaufbeuren, Germany). Specifically, we recorded data for ΔPO_2_, which represents the difference in the oxygen partial pressure between these two locations. This metric provides valuable information on the efficiency of oxygen exchange in the lungs. We compared ΔPO_2_ values across different groups and time intervals. In addition to ΔPO_2_, we also measured lactate levels. Lactate levels were measured as a biochemical indicator of metabolic stress and impaired oxygen utilization during EVLP.

### Tissue specimens

After the experiment, lung bronchial and alveolar tissues were acquired from diverse lung regions and fixed in 4% paraformaldehyde solution for 40 h. Then, the tissue underwent a gradual dehydration process, sequentially immersed in an ethanol to xylene gradient, meticulously embedded in paraffin, and finally sectioned at 6 μm for further research.

### Haematoxylin and eosin (H&E) staining

Paraffin-embedded slides were dewaxed with xylene and rehydrated with a decreasing ethanol series. Subsequently, the sections were stained with hematoxylin for 5 min, followed by eosin staining for 30 s. Following staining, the slides were dehydrated again with ethanol and xylene before being mounted using Mowiol® and coverslips.

#### Immunofluorescence staining

Paraffin sections were dewaxed and rehydrated as previously described, incubated with pepsin for 30 min to retrieve the antigen, and 10 min with 0.1% Triton-X100 supplied with PBS to permeabilize the cell membrane. The sections were then rinsed with water and phosphate-buffered saline (PBS). To prevent non-specific binding, cells were blocked using a mixture of PBS and 5% fetal calf serum (FCS) for 30 min at room temperature. The anti-sphingosine (1:1000) and anti-*P. aeruginosa* antibodies were diluted in 1% FCS in PBS and incubated with the samples for 45 min. The samples were then washed three times in PBS containing 0.05% Tween 20, followed by a single wash in pure PBS. Subsequently, the tissue was incubated with FITC-anti-mouse and Cy3-anti-rabbit antibodies diluted 1:500 with 1% FCS in PBS for 30 min. After another three washes in PBS with 0.05% Tween 20 and a final wash in PBS, samples were stained with DAPI. Examination was performed using a confocal microscope.

#### Histological evaluation

Paraffin-embedded tissue sections were evaluated based on the acute lung injury scoring methodology recommended by the American Thoracic Society^24^. The scoring criteria encompassed distinct histopathological features, including hyaline membranes, accumulation of proteinaceous exudate within the alveoli, increased alveolar septal thickness, and regions manifesting atelectasis.

#### Quantification of sphingosine and related metabolites by liquid chromatography tandem-mass spectrometry (LC–MS/MS)

Sphingosine and related sphingolipid metabolites were quantified by LC–MS/MS using previously established protocols for sphingolipid extraction and mass-spectrometric analysis, with minor modifications^25,26^. BALF samples (2 mL) and homogenates of human lung specimens (equivalents of 1 mg tissue) were subjected to lipid extraction using either 1-butanol (BALF) or methanol/chloroform (2:1, v:v, tissue homogenates). In both cases, the extraction solvent contained d_7_-sphingosine (d_7_-Sph), d_7_-sphingosine 1-phosphate (d_7_-S1P), C17 ceramide (C17 Cer) and d_31_-C16 sphingomyelin (d_31_-C16 SM) (all Avanti Polar Lipids, Alabaster, USA) as internal standards. Chromatographic separations were achieved on a 1290 Infinity II HPLC (Agilent Technologies, Waldbronn, Germany) equipped with a Poroshell 120 EC-C8 column (3.0 × 150 mm, 2.7 µm; Agilent Technologies). LC–MS/MS analyses were carried out using a 6495C triple-quadrupole mass spectrometer (Agilent Technologies) operating in the positive electrospray ionization mode (ESI +). Sph, S1P, Cer and SM were quantified by multiple reaction monitoring (qualifier product ions in parentheses): *m/z* 300.3 → 282.3 (252.3) for Sph, *m/z* 380.3 → 264.3 (82.1) for S1P, [M-H_2_O + H]^+^  → *m/z* 264.3 (282.3) for all Cer and [M + H]^+^  → *m/z* 184.1 (86.1) for all SM subspecies (C16, C18, C20, C22, C24 and C24:1). Peak areas of Cer and SM subspecies, as determined with MassHunter Quantitative Analysis software (version 10.1, Agilent Technologies), were normalized to those of the internal standards (C17 Cer or d_31_-C16 SM) followed by external calibration in the range of 1 fmol to 50 pmol on column. Sph and S1P were directly quantified via their deuterated internal standards d_7_-Sph (0.25 pmol on column) and d_7_-S1P (0.50 pmol on column). Determined sphingolipid amounts were normalized to sample volume (BALF) or the actual protein content (as determined by Bradford assay) of the tissue homogenate used for extraction.

#### Analysis of the association of bacterial cardiolipin and sphingosine

The association between bacterial cardiolipin and sphingosine was analyzed using an immunoprecipitation-based approach adapted from a previously published study on sphingosine–cardiolipin interaction, with minor modifications^17^. *P. aeruginosa* strain 762 was cultured as above and grown to the early logarithmic growth phase by incubation in TSB for 60 min at 37 °C with constant agitation at 125 rpm. Bacteria were centrifuged, washed twice in HEPES/Saline (H/S; 132 mM NaCl, 20 mM HEPES [pH 7.0], 5 mM KCl, 1 mM CaCl_2_, 0.7 mM MgCl_2_, 0.8 mM MgSO_4_) and 10^9^ CFU were incubated in a volume of 2.5 mL H/S with sphingosine at 5 μM final concentration. Sphingosine was suspended at 2.5 mM concentration in 0.9% NaCl as stock. The stock was thawed, sonicated in a bath sonicator for 10 min and then immediately added to the bacteria. Controls were treated with 0.9% NaCl only. Bacteria were incubated with sphingosine for 15 min, then centrifuged at 1700 g for 10 min, washed three times in H/S to remove free sphingosine and then lysed in TN3-buffer consisting of 125 mM NaCl, 10 mM EDTA, 25 mM TrisHCl (pH 7.4), 10 mM sodium pyrophosphate, 3% NP40 (= Igepal) and each 10 μg/mL aprotinin/leupeptin for 5 min on ice. Samples were centrifuged for 5 min at 20,800 g, the supernatants were collected and transferred into a new Eppendorf tube. An aliquot was employed to determine protein concentrations. The supernatants were then incubated for 30 min with 2 μg/mL anti-cardiolipin antibodies (USBiologicals, #C1375). Controls were incubated with an irrelevant human IgG. We then added 30 μL protein A/G agarose (Santa Cruz Inc., sc2003), incubated the samples for an additional 30 min at 4 °C with constant agitation, washed the samples four times in TN3 buffer and twice in H/S. The samples were finally centrifuged for 1 min at 20,800 g, the supernatant removed, the pellet resuspended in 200 μL H_2_O and immediately extracted in 600 μL CHCl_3_:CH_3_OH:1N HCl (100:100:1, v/v/v). Samples were sonicated, centrifuged for 5 min at 20,800 g to promote phase separation and an aliquot of the lower phase was dried in a Speedvac. The samples were resuspended in 20 μL of a detergent solution consisting of 7.5% [w/v] n-octyl glucopyranoside, 5 mM cardiolipin in 1 mM diethylenetriaminepentaacetic acid, immediately sonicated in a bath sonicator for 10 min and the kinase reaction was initiated by addition of 80 μL of 0.001 units sphingosine kinase in 50 mM HEPES (pH 7.4), 250 mM NaCl, 30 mM MgCl_2_, 1 mM ATP and 10 μCi [^32^P]γATP. The kinase reaction was performed for 30 min. Samples were extracted by addition of 20 μL 1N HCl, 800 μL CHCl_3_:CH_3_OH:1N HCl (100:200:1, v/v/v), and 240 μL each of CHCl_3_ and 2 M KCl. Samples were vortexed, phases separated, the lower phase was collected, dried, dissolved in 20 μL CHCl_3_:CH_3_OH (1:1, v/v), and separated on Silica G60 TLC plates with CHCl_3_:CH_3_OH:acetic acid:H_2_O (90:90:15:5, v/v/v/v) as developing solvent. The TLC plates were analyzed with a phosphorimager. Sphingosine levels were determined with a standard curve of C18-sphingosine.

#### Statistical analysis

Statistical analyses were performed using GraphPad Prism 9 software. Bronchial CFU counts were analyzed using two-way repeated-measures ANOVA, with treatment group as the between-subject factor and time (before versus after treatment) as the within-subject factor, followed by Šídák’s multiple-comparison test. Paired two-tailed Student’s t-tests were used for other paired comparisons, including comparisons between matched treated and untreated human lung regions. Unpaired two-tailed Student’s t-tests were used for comparisons between two independent groups. Comparisons among more than two independent groups were performed using one-way ANOVA followed by Tukey’s multiple-comparison test. For physiological and biochemical parameters recorded repeatedly during EVLP, data are presented over time for descriptive purposes, whereas statistical comparisons between groups were based on predefined summary measures calculated for each individual lung, including treatment-associated changes in mean values and differences between hour 1 and hour 4 of perfusion. This approach was used to avoid treating repeated time-point measurements as independent observations in the setting of a small EVLP sample size. All tests were two-sided, and a P value < 0.05 was considered statistically significant.

## Results

### Sphingosine reduces P. aeruginosa burden in the bronchial epithelium

**Fig. 2:**
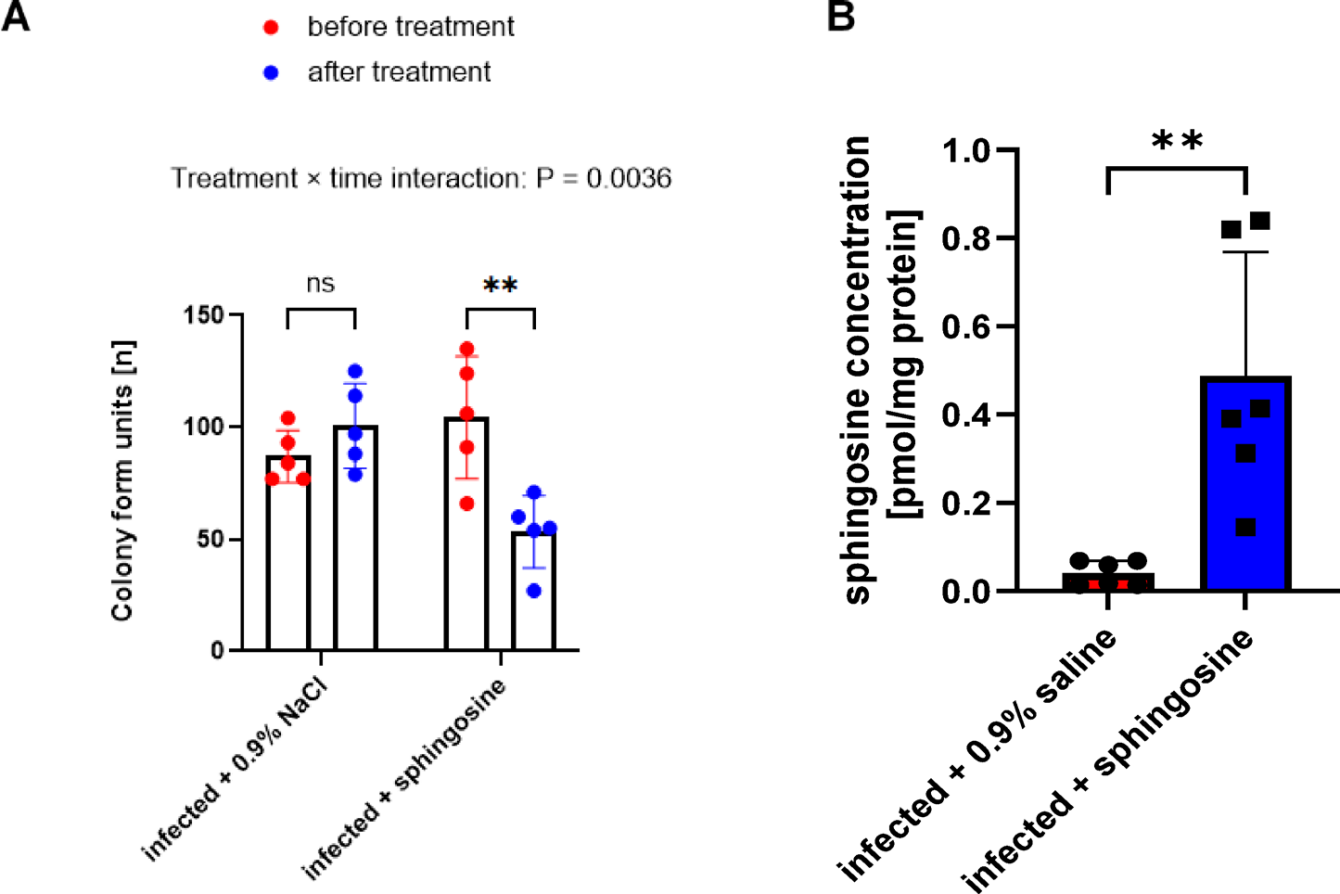
Sphingosine reduces bacterial burden and associates with cardiolipin in *P. aeruginosa*. **A** Bronchial biopsy samples were collected from infected lungs immediately before and after treatment with either 0.9% NaCl or sphingosine. Inhalation of 0.9% NaCl did not reduce the CFU counts of P. aeruginosa strain 762, whereas inhalation of sphingosine significantly reduced bacterial burden compared with the corresponding pretreatment values. Each data point represents one independent EVLP experiment. Data are presented as mean ± SD. Statistical analysis was performed using two-way repeated-measures ANOVA followed by Šídák’s multiple-comparison test. **B** Immunoprecipitation of cardiolipin revealed its association with sphingosine after treatment of *P. aeruginosa* strain 762 with sphingosine for 15 min. Bacteria were incubated with sphingosine, lysed, and cardiolipin was immunoprecipitated; associated sphingosine was then determined using a kinase assay. Sphingosine concentrations were normalized to protein concentrations in the bacterial lysates. Control lysates were incubated with irrelevant human IgG, and the background values were subtracted from the specific signal. Data are shown as mean ± SD from 5 independent experiments. Statistical analysis was performed using Student’s t-test. **P < 0.01; ns, not significant.

To investigate the effect of inhaled sphingosine on *P. aeruginosa* strain 762, we performed biopsies directly from the bronchial epithelium and used saponin to permeabilize the tissue. The number of CFUs represents the combined amount of intracellular and extracellular bacterial cells. A two-way repeated-measures ANOVA demonstrated a significant treatment × time interaction for bronchial bacterial counts (F(1,8) = 16.48, P = 0.0036), indicating that the change in CFU over time differed significantly between the sphingosine and 0.9% NaCl groups. Post hoc Šídák multiple-comparison analysis showed that sphingosine treatment significantly reduced CFU counts (P = 0.0038), whereas no significant change was observed in the 0.9% NaCl group (P = 0.4542) (Fig. 2A).

We have previously shown that sphingosine induces degradation of cardiolipin in P. aeruginosa, thereby mediating bacterial death^17^. To explore whether sphingosine associates with cardiolipin in P. aeruginosa strain 762, we performed cardiolipin immunoprecipitation after sphingosine exposure. Sphingosine signal was detected in cardiolipin immunoprecipitates after sphingosine treatment, consistent with previous reports describing sphingosine-cardiolipin interaction (Fig. 2B).

### The effect of *P. aeruginosa* infection and sphingosine inhalation on lung function

**Fig. 3:**
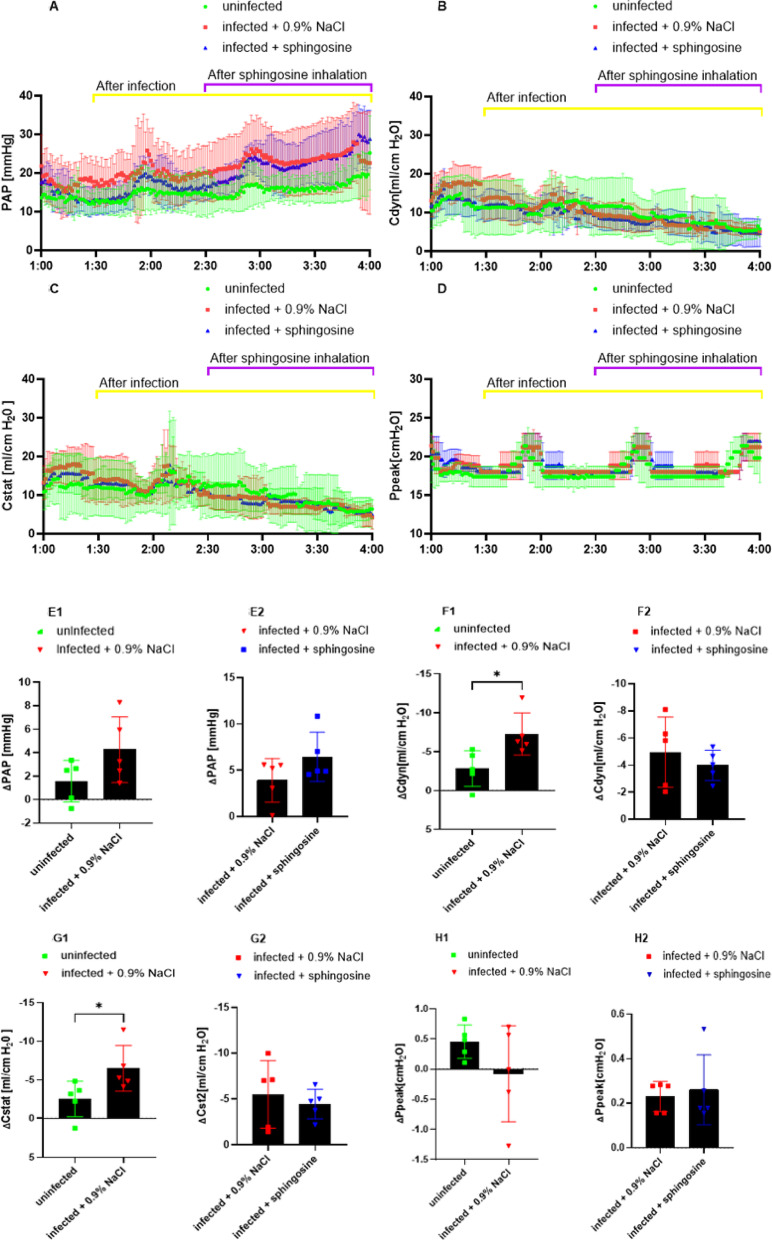
Changes in lung physiological parameters during EVLP. Mean values of pulmonary artery pressure (PAP), dynamic compliance (Cdyn), static compliance (Cstat), and peak airway pressure (Ppeak) were recorded during perfusion (**A**–**D**). Group comparisons were based on predefined summary measures. Repeated measurements were averaged within predefined time intervals for each lung, and per-lung change values were used for statistical analysis. Changes in mean values before and after the first intervention, namely *P. aeruginosa* inhalation in infected lungs or 0.9% NaCl inhalation in uninfected lungs, are shown as ΔPAP1, ΔCdyn1, ΔCstat1, and ΔPpeak1 (E1–H1). Changes in mean values before and after the second intervention, namely sphingosine inhalation in the infected + sphingosine group or 0.9% NaCl inhalation in the control groups, are shown as ΔPAP2, ΔCdyn2, ΔCstat2, and ΔPpeak2 (E2–H2). Data are presented as mean ± SD (n = 5 per group). Comparisons between independent groups were performed using unpaired two-tailed t-tests. Exact P values are reported in the Results section. *P < 0.05.

The data for pulmonary artery pressure (PAP), dynamic compliance (Cdyn), static compliance (Cstat), and peak airway pressure (Ppeak) during perfusion are shown in Fig. 3A–D. For group comparisons, repeated measurements were averaged within predefined time intervals for each individual lung, and per-lung change values were used for statistical analysis. The impact of *P. aeruginosa* infection on these parameters was evaluated by comparing changes after the first intervention between uninfected lungs and infected lungs treated with 0.9% NaCl (Fig. 3E1, F1, G1, H1). Compared with uninfected lungs, infected lungs treated with 0.9% NaCl exhibited a greater decline in dynamic compliance (P = 0.0235) and static compliance (P = 0.0468), whereas changes in PAP (P = 0.1141) and Ppeak (P = 0.2166) did not reach statistical significance. To assess the effect of sphingosine on lung mechanics, changes after the second intervention were compared between infected lungs treated with sphingosine and infected lungs treated with 0.9% NaCl (Fig. 3E2, F2, G2, H2). No statistically significant differences were observed between the two groups for PAP (P = 0.1483), Cdyn (P = 0.4745), Cstat (P = 0.5845), or Ppeak (P = 0.7105), suggesting that sphingosine inhalation did not measurably worsen lung function under the present 4-h EVLP conditions.

### Blood gas analysis

**Fig. 4:**
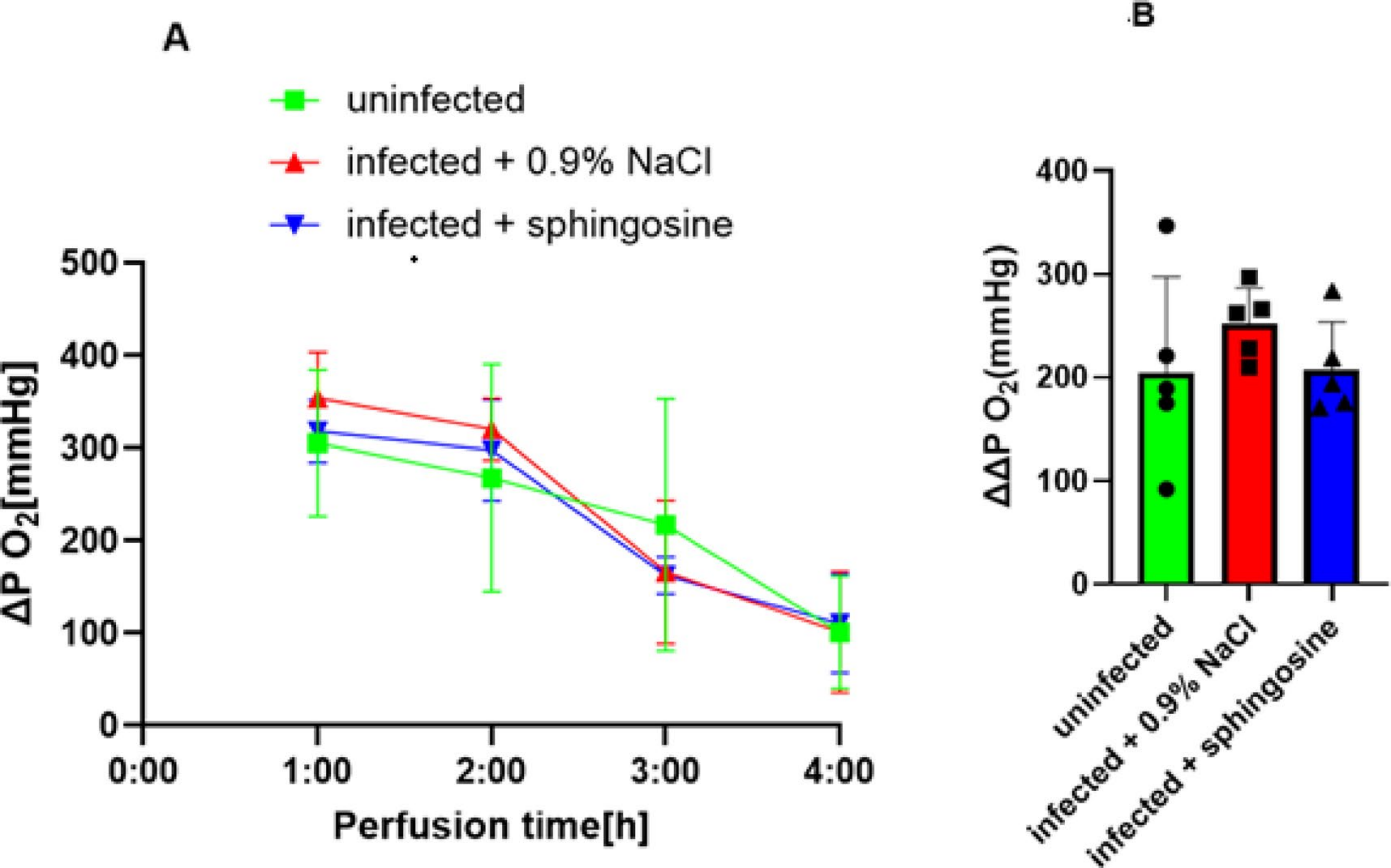
Blood gas analysis during EVLP. **A** ΔPO₂ represents the difference in partial pressure of oxygen between the pulmonary vein and pulmonary artery and reflects pulmonary oxygen exchange capacity. ΔPO₂ was recorded hourly during perfusion. **B** ΔΔPO₂ denotes the change in ΔPO₂ between the first and fourth hour of perfusion. Data are presented as mean ± SD. Statistical analysis for group comparisons was performed using one-way ANOVA followed by Tukey’s multiple-comparison test. Exact P values are reported in the Results section.

**Fig. 5:**
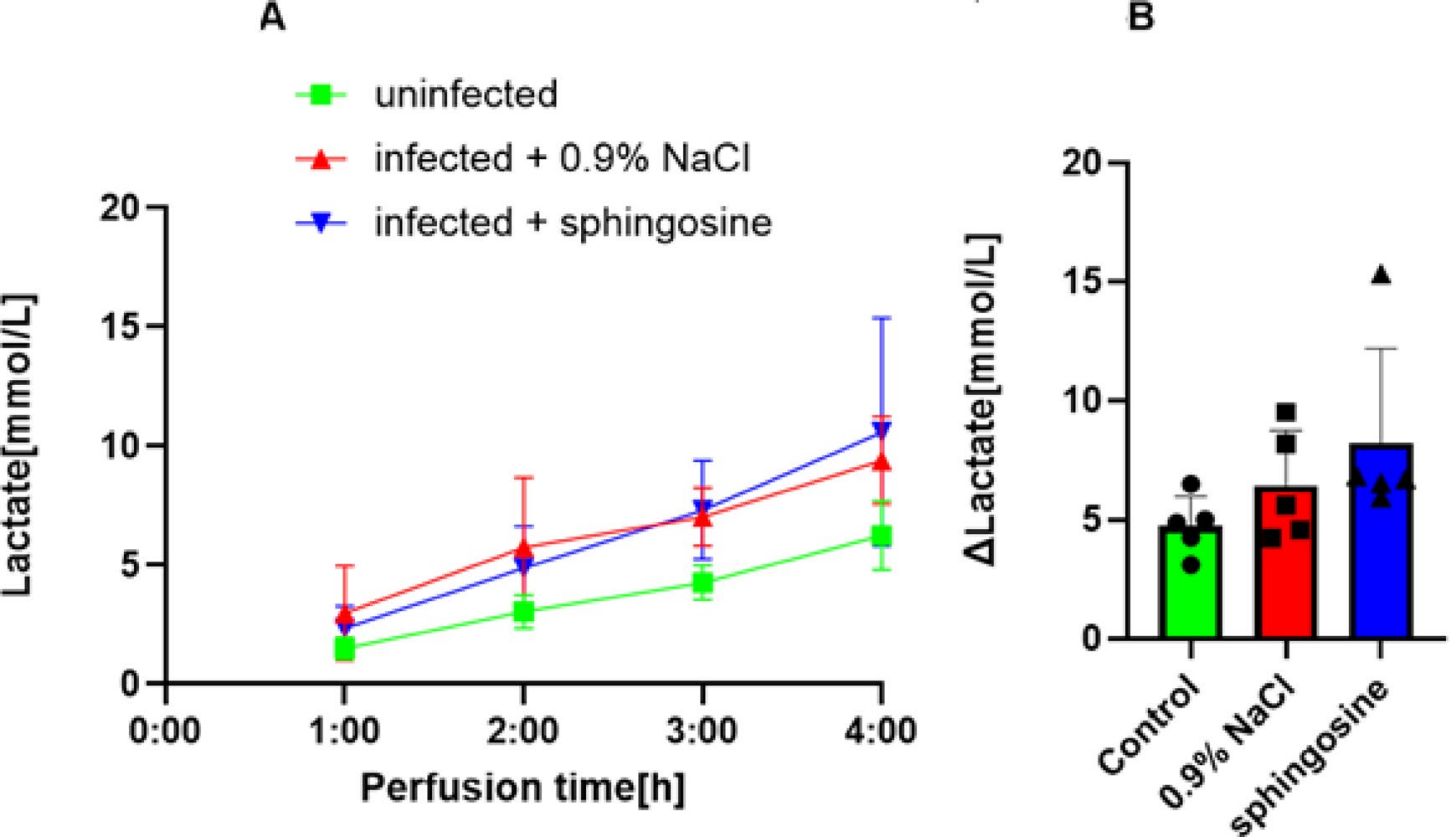
Lactate levels in the perfusate during EVLP. **A** Lactate concentrations were measured hourly during perfusion. Lactate levels increased progressively over time in all groups. Data are shown as mean ± SD. **B** ΔLactate represents the change in lactate concentration between the first and fourth hours of perfusion, reflecting alterations in tissue hypoxia during this period. Statistical comparisons among groups were performed using one-way ANOVA followed by Tukey’s multiple-comparison test.

ΔPO₂ values appeared descriptively similar among the groups at each time point during perfusion (Fig. 4A). Throughout perfusion, ΔPO₂ gradually decreased in all groups, indicating a progressive decline in oxygen exchange capacity. The change in ΔPO₂ between the first and fourth hours of perfusion, defined as ΔΔPO₂, did not differ significantly among the groups (uninfected vs. infected + 0.9% NaCl, P = 0.4747; uninfected vs. infected + sphingosine, P = 0.9969; infected + 0.9% NaCl vs. infected + sphingosine, P = 0.5171; Fig. 4B). Lactate levels increased progressively over time in all groups during perfusion (Fig. 5A). ΔLactate, defined as the change in lactate concentration between the first and fourth hours of perfusion, did not differ significantly among the groups (uninfected vs. infected + 0.9% NaCl, P = 0.6117; uninfected vs. infected + sphingosine, P = 0.1526; infected + 0.9% NaCl vs. infected + sphingosine, P = 0.5652; Fig. 5B).

### Immunofluorescence staining of *P. aeruginosa* and sphingosine

**Fig. 6:**
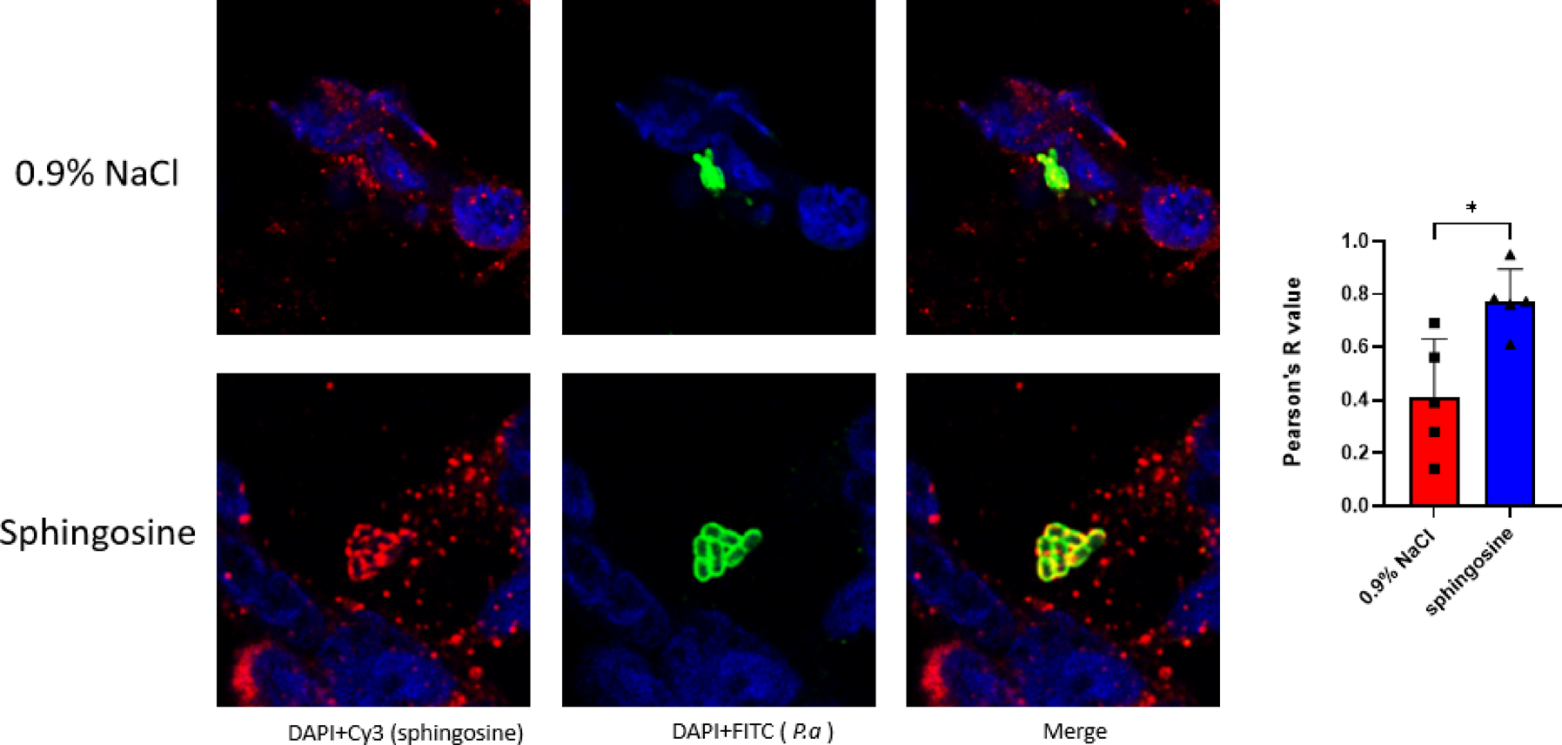
Immunofluorescence staining of P. aeruginosa and sphingosine. Paraffin sections of lung parenchymal tissue from infected lungs treated with 0.9% NaCl or sphingosine, as indicated on the Y axis, were stained with FITC-conjugated anti-P. aeruginosa antibodies (green) and Cy3-conjugated anti-sphingosine antibodies (red). Cell nuclei were counterstained with DAPI (blue). Merged images show colocalization of sphingosine and P. aeruginosa as yellow signals. The bar graph shows quantification of colocalization using Coloc2 analysis. Data are presented as mean ± SD. Statistical analysis was performed using Student’s t-test. *P < 0.05.

Colocalization of sphingosine and *P. aeruginosa* was observed in both infected lungs treated with 0.9% NaCl and those treated with sphingosine, as shown in Fig. 6. Notably, quantitative Coloc2 analysis revealed increased colocalization following sphingosine treatment (P < 0.05), indicating enhanced association of sphingosine with *P. aeruginosa*.

### Histological assessment of lung tissue

**Fig. 7:**
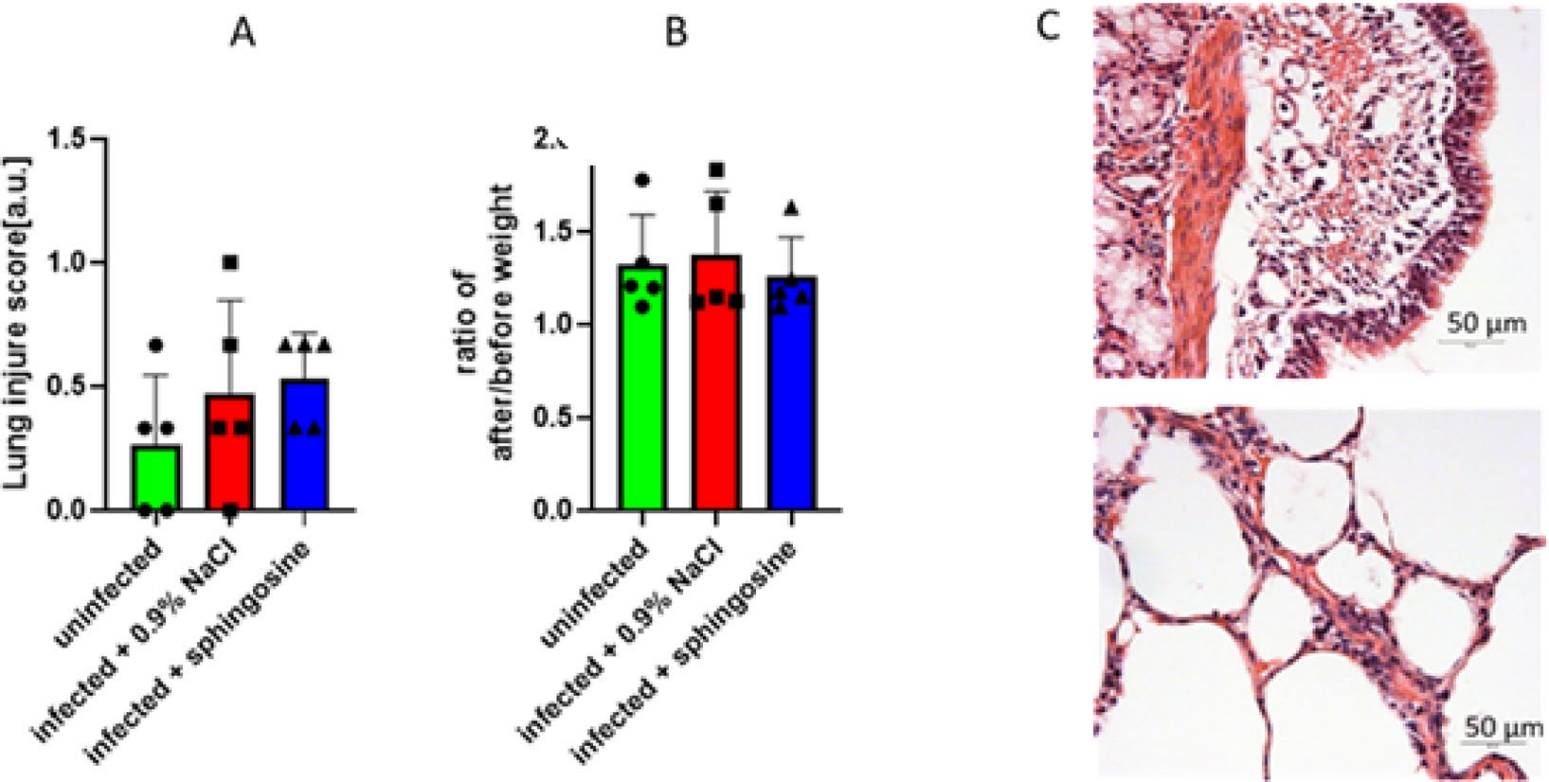
Histology assessment and lung weight after EVLP. Paraffin-embedded lung sections were stained with haematoxylin and eosin (H&E) and histological injury was evaluated using a standardized lung injury scoring system (**A**). Lung weights were recorded before and after EVLP, and lung weight ratios were calculated and compared between groups (**B**). Representative H&E-stained images are shown in panel C. Data are presented as mean ± SD (n = 5). Statistical analysis was performed using one-way ANOVA followed by Tukey’s multiple-comparison test, and exact P values are reported in the Results.

Upon microscopic examination of pulmonary tissue sections, no marked epithelial layer disruption, leukocyte infiltration, or damage to the alveolar–capillary membrane was observed in any of the experimental groups (Fig. 7C). Quantitative lung injury scores did not differ significantly between uninfected lungs and infected lungs treated with 0.9% NaCl (P = 0.5417), uninfected lungs and infected lungs treated with sphingosine (P = 0.3504), or between the two infected groups (P = 0.9310) (Fig. 7A).

### Lung weight gain

Lung weight gain is a key indicator of pulmonary edema formation following perfusion. Across all groups, lung weight increased after 4 h of EVLP (Fig. 7B). However, comparison of lung weight ratios before and after perfusion revealed no statistically significant differences between uninfected lungs and infected lungs treated with 0.9% NaCl (P = 0.9520), uninfected lungs and infected lungs treated with sphingosine (P = 0.9298), or between the two infected groups (P = 0.7881).

### Sphingosine inhalation increases tissue sphingosine levels and alters sphingolipid metabolites in explanted human lungs

To explore whether inhaled sphingosine can be delivered to human lung tissue, ex vivo experiments were performed using explanted human recipient lungs. Following sphingosine inhalation, concentrations of sphingosine, sphingosine-1-phosphate (S1P), ceramide, and sphingomyelin were quantified in bronchoalveolar lavage fluid (BALF), bronchial epithelial tissue, and lung parenchyma by LC–MS/MS. For bronchial epithelial and lung parenchymal analyses, two tissue subsamples obtained under each condition were averaged to generate one value for each of the four lungs.

**Fig. 8:**
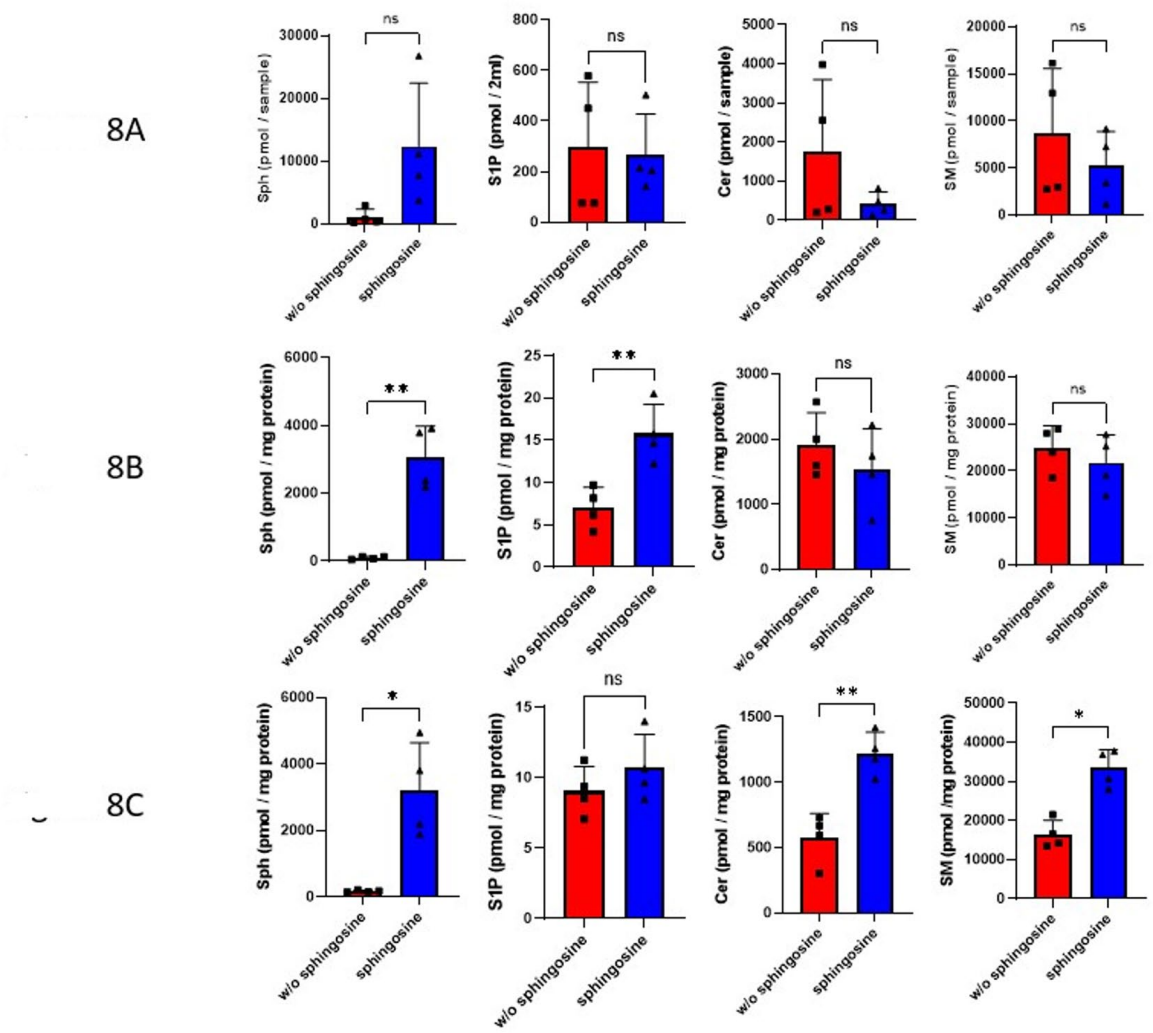
Sphingosine inhalation alters sphingolipid metabolism in human lung tissues. Sphingolipids were quantified by LC–MS/MS in BALF (**A**), bronchial epithelial tissue (**B**), and lung parenchymal tissue (**C**) from four explanted human lungs under paired control and sphingosine inhalation conditions. For bronchial epithelial and parenchymal tissues, two tissue subsamples were obtained from each lung under each condition and averaged to generate one value per lung. Each data point represents one lung (*n=4* biologically independent lungs). Sphingosine levels increased following inhalation, with statistically significant increases in bronchial epithelial and parenchymal tissues. Data are presented as mean ± SD. Paired samples obtained from the same lungs were analyzed using two-tailed paired Student’s t-tests. *P < 0.05, **P < 0.01; ns, not significant.

LC–MS/MS analysis showed that sphingosine inhalation altered sphingolipid levels in a tissue-specific manner. Although BALF sphingosine levels were numerically increased following inhalation, the difference was not statistically significant. No significant changes in BALF S1P, ceramide, or sphingomyelin levels were observed (Fig. 8A). In bronchial epithelial tissue, sphingosine (P = 0.0064) and S1P (P = 0.0069) levels were significantly increased, whereas ceramide and sphingomyelin levels remained unchanged (Fig. 8B). In lung parenchymal tissue, sphingosine (P = 0.0250), ceramide (P = 0.0012), and sphingomyelin (P = 0.0126) levels were significantly increased, while S1P levels did not differ significantly between the paired conditions (Fig. 8C).

**Fig. 9:**
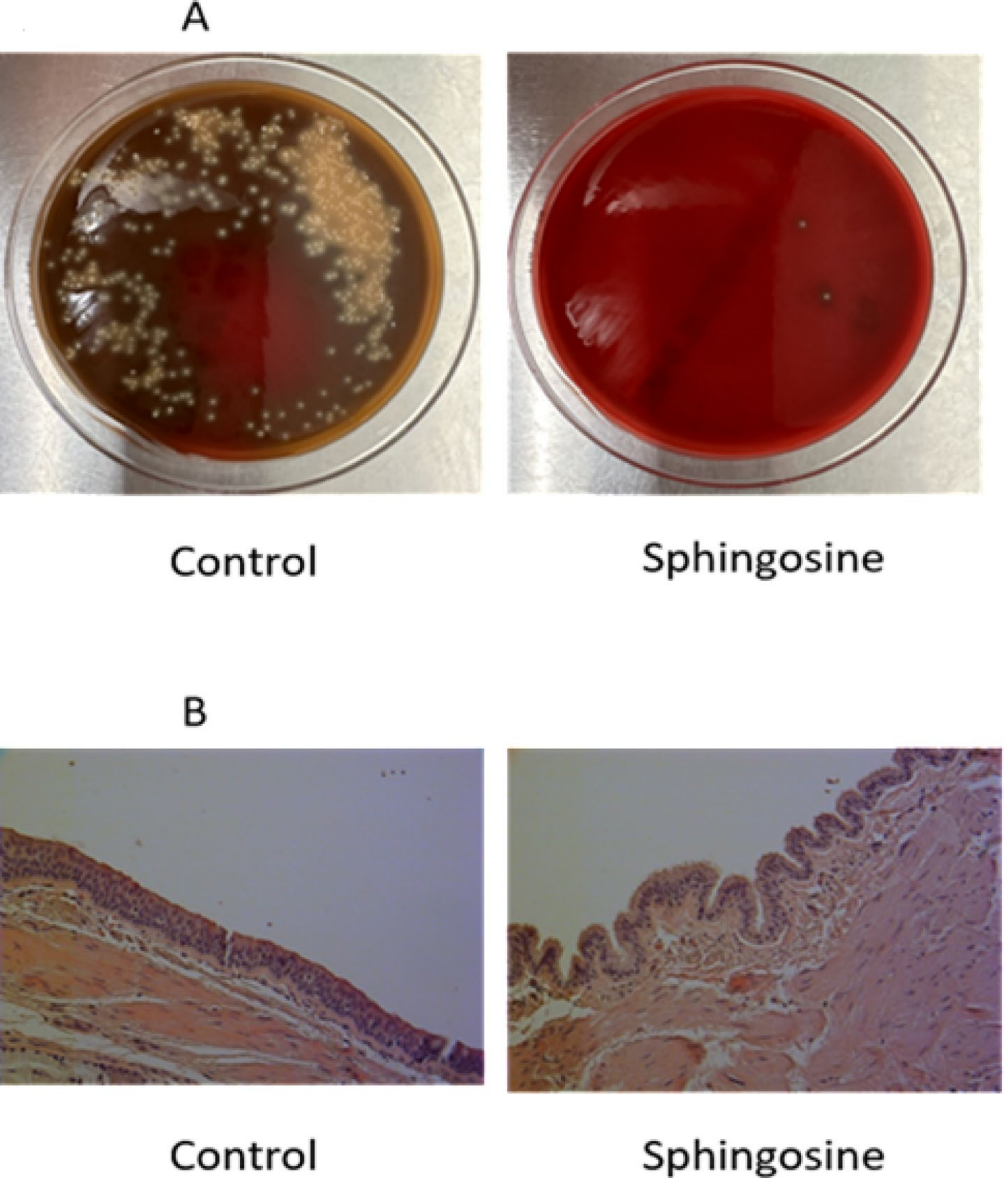
Antibacterial effect and histological tolerability of inhaled sphingosine in explanted human lungs. **A** Representative images of bacterial growth on blood agar plates from bronchoalveolar lavage fluid (BALF) samples obtained before and after sphingosine inhalation. In one explanted recipient lung specimen with pre-existing bacterial colonization, substantial bacterial growth was observed before sphingosine inhalation, while sphingosine treatment led to near-complete elimination of detectable bacteria. **B** Representative histological sections of bronchial epithelial tissue stained with hematoxylin and eosin (H&E). No structural damage or epithelial disruption was observed after sphingosine inhalation, indicating that inhaled sphingosine did not adversely affect tissue integrity under these short-term ex vivo conditions.

Among the four explanted human lungs included in the study, bacterial colonization was detected in one specimen during microbiological assessment (Fig. 9A). In this single colonized lung, detectable bacterial growth was markedly reduced after sphingosine inhalation. Because this observation was limited to one specimen, it was considered exploratory and hypothesis-generating. Furthermore, histological analysis of bronchial epithelial cells revealed no apparent structural damage following sphingosine inhalation (Fig. 9B), indicating good local tolerability under these ex vivo conditions.

## Discussion

P. aeruginosa is a concern in the medical context because of its multidrug resistance capabilities and frequent infections, notably in lung transplant recipients^2,4,7,27^. The present study evaluated sphingosine as a locally delivered antibacterial strategy for P. aeruginosa airway contamination in a pre-transplant EVLP setting. Our examination of inhaled sphingosine’s effects on P. aeruginosa-infected ex vivo perfused porcine lungs yielded promising results. We observed a substantial reduction in bacterial burden of the bronchial epithelium. Because no systemic antibiotics were administered during EVLP, this reduction was not attributable to concomitant antimicrobial treatment in the perfusate. These findings are consistent with previous literature, suggesting that sphingosine has a robust antibacterial capability against P. aeruginosa both in vitro and in vivo, highlighting the potential of sphingosine as a local antimicrobial approach during EVLP^22^.

During EVLP, we also focused on evaluating the impact of P. aeruginosa infection and sphingosine treatment on lung functions by assessing lung function metrics, specifically PAP, Cdyn, Cstat, and Ppeak. Shifts in these metrics post-infection or post-treatment are clinically relevant, with changes potentially indicating the degree of lung injury and the efficacy of the P. aeruginosa strain used^28^. Additionally, any improvement following sphingosine administration might indicate its therapeutic utility. The findings revealed that Cdyn and Cstat experienced a greater decline in the infected group than in the uninfected counterpart post-infection, but sphingosine treatment did not reverse this decline. The absence of measurable improvement in lung function may be attributable to the short experimental duration, which may have been insufficient to allow functional recovery following the reduction in bacterial burden. Thus, although the precise mechanism was not explored in this study, it is possible that the initial damage by P. aeruginosa before sphingosine introduction influenced the outcomes in these short-term studies. The short experimental period may also explain the absence of significant differences in lung oxygen exchange, histological changes, and lactate levels between the uninfected and infected groups.

Our findings indicated that sphingosine inhalation did not induce any notable acute changes in lung function during the 4-h EVLP period. Thus, the present data support short-term local tolerability under these experimental conditions, but they should not be interpreted as definitive long-term safety data. Delayed toxicity, inflammatory responses after longer exposure, post-transplant graft function, and recipient outcomes will require further investigation.

Our data showed co-localization of sphingosine with P. aeruginosa upon inhalation of sphingosine. It is likely that endogenous sphingosine formed upon infection also co-localizes with the bacteria and that the effects of endogenous sphingosine on the pathogens are enhanced by inhalation of sphingosine. This suggests that the anti-bacterial effects of sphingosine require a certain concentration of sphingosine in the bacteria, consistent with previous publications^15,16,29,30^. Reduced airway sphingosine levels have also been linked to increased susceptibility to bacterial infection in cystic fibrosis, where β1-integrin accumulation in luminal airway epithelial membranes decreases sphingosine levels^31^. Mechanistically, sphingosine-mediated killing of *P. aeruginosa* has been shown to involve degradation of bacterial cardiolipin through the maintenance of outer lipid asymmetry system^32^. Sphingosine can also kill intracellular *P. aeruginosa* and *Staphylococcus aureus*^33^. Together with our previous finding that inhaled sphingosine reduced *S. aureus* infection in ex vivo perfused and ventilated lungs^34^, these observations support the broader potential of sphingosine as a local antimicrobial strategy in infected lungs.

In the human lung experiments, we demonstrated that inhaled sphingosine could be effectively delivered to and detected in human lung tissue, including both bronchial epithelial tissue and the lung parenchyma. The increases in sphingosine, ceramide, and sphingomyelin levels in the parenchymal compartment are consistent with local remodeling of sphingolipid metabolism following sphingosine exposure. Interestingly, S1P levels increased selectively in bronchial epithelial tissue, whereas ceramide and sphingomyelin levels remained unchanged in this compartment. These findings suggest that inhaled sphingosine induces distinct, tissue-specific sphingolipid responses in different lung compartments, highlighting the complexity of sphingolipid metabolism in human lung tissue. Further studies are required to elucidate the regulatory pathways and enzymatic mechanisms underlying these tissue-specific metabolic changes.

In the single colonized human lung specimen, the marked reduction in detectable bacterial growth after sphingosine inhalation, without apparent histological damage, supports further investigation of sphingosine under clinically relevant ex vivo conditions. However, because this observation was limited to one specimen, it should be interpreted as exploratory and hypothesis-generating rather than evidence of antibacterial efficacy in human lungs.

However, this study has several limitations. First, the EVLP observation period was limited to 4 h. This duration was sufficient to assess acute changes in bacterial burden and short-term physiological effects but does not allow evaluation of delayed toxicity or post-transplant outcomes. Second, an internal uninfected sphingosine-treated EVLP group was not included, as inhaled sphingosine has previously been evaluated in non-infected isolated ventilated and perfused pig lungs without relevant adverse effects^21^. Third, bronchial CFU counts were expressed per biopsy sample rather than normalized to total tissue weight, because biopsy weight may be strongly affected by variable amounts of cartilage and submucosal tissue and may therefore not accurately reflect the amount of bronchial epithelium sampled. Nevertheless, minor variation in the epithelial area sampled cannot be fully excluded. Fourth, only a single P. aeruginosa strain was used, which may not represent the full spectrum of bacterial susceptibility. Future studies, beyond the focus of the present study, should include a broader range of clinical isolates, especially multidrug-resistant strains. Fifth, in the human lung experiments, histological evaluation was limited to bronchial tissue. Since the recipient lungs used in this study were explanted from patients with underlying conditions such as emphysema or pulmonary fibrosis, analysis of parenchymal histological changes was limited by pre-existing pathology. Moreover, the explanted recipient lungs had severely damaged vasculature, which prevented connection to a perfusion circuit and limited the experiments to ventilation only. Finally, the human microbiological observation was limited to one colonized specimen; therefore, the human data should be regarded as exploratory and hypothesis-generating.

In summary, inhaled sphingosine reduced bronchial P. aeruginosa burden in a porcine EVLP model of acute airway contamination without detectable short-term worsening of measured physiological, biochemical, histological, or edema-related parameters. Exploratory experiments in explanted human recipient lungs demonstrated delivery of sphingosine to bronchial and parenchymal tissue and no apparent acute histological damage in assessed airway samples. Because the human microbiological observation was limited to one colonized specimen, these data should be interpreted as hypothesis-generating. Further studies using colonized donor lungs, clinically relevant multidrug-resistant respiratory isolates, antibiotic comparators, and longer observation periods are required to determine the potential role of sphingosine as a local antimicrobial strategy during EVLP.

## Data Availability

The data that support the findings of this study are available from the corresponding author upon reasonable request.

## Abbreviations

EVLP: Ex vivo Lung perfusion
*P. aeruginosa*: *Pseudomonas aeruginosa*
CFU: Colony-forming units
PAP: Pulmonary artery pressure
Cdyn: Dynamic lung compliance
Cstat: Static lung compliance
Ppeak: Peak airway pressure
HE: Haematoxylin–eosin
PBS: Phosphate-buffered saline
FCS: Fetal calf serum
TSA: Tryptic soy agar
OD: Optical density
ANOVA: Analysis of variance
